# Parkinson's Disease Exhibits Amplified Intermuscular Coherence During Dynamic Voluntary Action

**DOI:** 10.3389/fneur.2020.00204

**Published:** 2020-04-03

**Authors:** Christopher M. Laine, Francisco J. Valero-Cuevas

**Affiliations:** ^1^Division of Biokinesiology and Physical Therapy, University of Southern California, Los Angeles, CA, United States; ^2^Department of Biomedical Engineering, University of Southern California, Los Angeles, CA, United States

**Keywords:** coherence, EMG, biomarker, manual tasks, alpha-band, kinetic tremor, action tremor

## Abstract

Parkinson's disease (PD) is typically diagnosed and evaluated on the basis of overt motor dysfunction, however, subtle changes in the frequency spectrum of neural drive to muscles have been reported as well. During dynamic actions, coactive muscles of healthy adults often share a common source of 6–15 Hz (alpha-band) neural drive, creating synchronous alpha-band activity in their EMG signals. Individuals with PD commonly exhibit kinetic action tremor at similar frequencies, but the potential relationship between the intermuscular alpha-band neural drive seen in healthy adults and the action tremor associated with PD is not well-understood. A close relationship is most tenable during voluntary dynamic tasks where alpha-band neural drive is strongest in healthy adults, and where neural circuits affected by PD are most engaged. In this study, we characterized the frequency spectrum of EMG synchronization (intermuscular coherence) in 16 participants with PD and 15 age-matched controls during two dynamic motor tasks: (1) rotation of a dial between the thumb and index finger, and (2) dynamic scaling of isometric precision pinch force. These tasks produce different profiles of coherence between the first dorsal interosseous and abductor pollicis brevis muscles. We sought to determine if alpha-band intermuscular coherence would be amplified in participants with PD relative to controls, if such differences would be task-specific, and if they would correlate with symptom severity. We found that relative to controls, the PD group displayed amplified, but similarly task-dependent, coherence in the alpha-band. The magnitude of coherence during the rotation task correlated with overall symptom severity as per the UPDRS rating scale. Finally, we explored the potential for our coherence measures, with no additional information, to discriminate individuals with PD from controls. The area under the Receiver Operating Characteristic curve (AUC) indicated a clear separation between groups (AUC = 0.96), even though participants with PD were on their typical medication and displayed only mild-moderate symptoms. We conclude that a task-dependent, intermuscular neural drive within the alpha-band is amplified in PD. Its quantification via intermuscular coherence analysis may provide a useful tool for detecting the presence of PD, or assessing its progression.

## Introduction

The evaluation of Parkinson's disease (PD) is currently dominated by subjective clinical ratings of symptom severity, such as the Unified Parkinson's Disease Rating Scale (UPDRS). The coarse nature of this examination, along with the diversity of possible symptoms, has driven a search for more direct, quantitative measures of neural dysfunction which can objectively assess the early presence and progression of the disease.

The frequency spectrum of neural activity within the motor system is altered in PD. Neural oscillations are ubiquitous in the healthy motor system, but PD is characterized by a particularly large variety of abnormal oscillations. Tremor at rest (rest tremor) typically has a frequency of ~3–6 Hz, while “action tremor” extends from ~6–15 Hz and occurs during voluntary static (postural action tremor) or dynamic (kinetic action tremor) muscle activation (1–6). Further, PD is associated with increased corticomuscular drive in the range of 15–30 Hz during static contractions (7–11), as well as reduced neural drive to muscles in the 30–50 Hz range in unmedicated patients (12).

During motor behavior, coactive muscles often share a portion of their neural drive which synchronizes their activities at different frequencies. This entrainment of muscle activity by a common source of oscillatory neural drive can be quantified in the frequency domain by calculating the coherence between their EMG signals (13–20). Intermuscular coherence associated with postural action tremor (21) or rest tremor (22) in PD suggests that the distribution of tremulous neural drive across muscles could be an important feature of the disease, and one which cannot be assessed reliably within the context of typical clinical evaluations. Clinical evaluations, and indeed most scientific studies, have focused on the visible/overt forms of tremor, but it has been known for decades that in PD, dynamic voluntary activity consistently evokes kinetic action tremor in the alpha-band (~6–15 Hz) which simultaneously affects multiple muscles and is observable in EMG even when no visible tremor is apparent (1).

In fact, this kinetic action tremor is the strongest and most consistently-evoked form of non-overt tremor in PD (1, 4, 23–25), and can be found in most individuals with PD. While kinetic action tremor is often described as an amplification of the ~6–15 Hz physiological tremor seen in healthy adults due to its similarity in frequency and the fact that this frequency doesn't change with loading (4, 26), its underlying neurophysiology in PD is not well-understood, especially since ~6–15 Hz neural drive to muscles can come from a variety of different sources (27–36). Also, compared with other manifestations of PD, kinetic action tremor has received relatively little attention.

Most previous studies of action tremor in PD have focused on forces, motions, or individual muscles rather than the coherence of tremor-generating drive across muscles. If the relevant descending drive is fundamentally intermuscular, then action tremor should not only depend on action, but also on the dynamic coordination among muscles required by a given task, as this is known to influence the strength of ~6–15 Hz intermuscular neural drive (16, 19, 32, 37, 38). While elevated intermuscular coherence between anatomically-synergistic muscles has been found during static voluntary tasks in PD (7, 11), these findings may not extend to dynamic actions, or to functionally-different muscles whose coordination can change depending on task.

The aim of this study was therefore to characterize intermuscular coherence during voluntary dynamic tasks in participants with PD, compared to age-matched controls. Specifically, we tested two tasks: (1) rotation of a dial between the thumb and index finger, and (2) dynamic scaling of isometric precision pinch force. These tasks were chosen because they evoke different levels of coherence (especially between 6 and 15 Hz) between the first dorsal interosseous (FDI) and abductor pollicis brevis (APB) muscles (19), and because dynamic multifinger manipulation with the fingertips evokes strong functional coupling among the fingers (39).

Our primary hypotheses were that (1) intermuscular coherence would be larger in PD, especially at ~10 Hz during both dynamic tasks, (2) the task-dependent modulation of intermuscular coherence seen in controls would be preserved in PD, and (3) the amplification of alpha-band coherence would correlate with clinical severity, since dynamic tasks should preferentially utilize neural pathways known to deliver alpha-band drive to muscles, and which are known to be disrupted in PD, such as the cerebello-thalamo-cortical circuit (30, 40–42). Given that intermuscular coherence analysis has been suggested as a potential avenue for biomarker development (11), and that we lack simple, cost-effective methods for detecting the presence of PD and the severity of neural damage, a secondary aim was to determine the ability of coherence measures to discriminate between PD and control groups, as this would justify future efforts to develop clinically-applicable metrics using EMG.

## Methods

### Participants

We recruited 16 individuals with mild-moderate severity Parkinson's disease (age: 62.3 +/– 8.6, 8 male) and 15 control participants (age: 60.5 +/– 10.3, 9 male). The details of the patient population are shown in Table 1. All participants with PD were on their normal medication, and in the ON state at the time of testing. All were diagnosed with idiopathic Parkinson's disease, and all but one were on dopaminergic medication. All participants understood the task and scored >23 on the mini mental status exam. Our sample size is intended to be sufficient for detection of large differences and correlations, and is in line with similar recent literature [e.g., Flood et al., (11)] where such effects were found.

**Table 1:** Patient characteristics.

**Patients**	**TSD**	**H&Y**	**UPDRS_total**	**UPDRS_II**	**UPDRS_III**	**LEDD**
1	2	2	29	4	14	764
2	5.5	2	31	6	8	200
3	8	2	31	10	13	216
4	6	2	47	9	29	400
5	2.5	2	23	5	4	400
6	2	2	25	10	7	150
7	2.5	2	13	4	5	840
8	2	2	23	1	22	300
9	3.5	2	51	11	12	300
10	4	2	23	11	8	400
11	6	2	51	17	24	400
12	7	2	42	9	17	0
13	1.5	1	15	0	6	512.5
14	0.5	2	10	1	8	1305
15	1	2	52	15	22	287.5
16	1	2	25	7	10	100

*TSD, Time Since Diagnosis (years); H&Y, Hoehn and Yahr; UPDRS_total, combined score on Unified Parkinson's Disease Rating Scale (UPDRS); UPDRS_II, Activities of daily living; UPDRS_III, Motor evaluation; LEDD, Levodopa Equivalent Daily Dose*.

All participants gave written informed consent prior to participation and all procedures were approved by the University of Southern California Institutional Review Board.

### Experimental Setup

**Figure 1:**
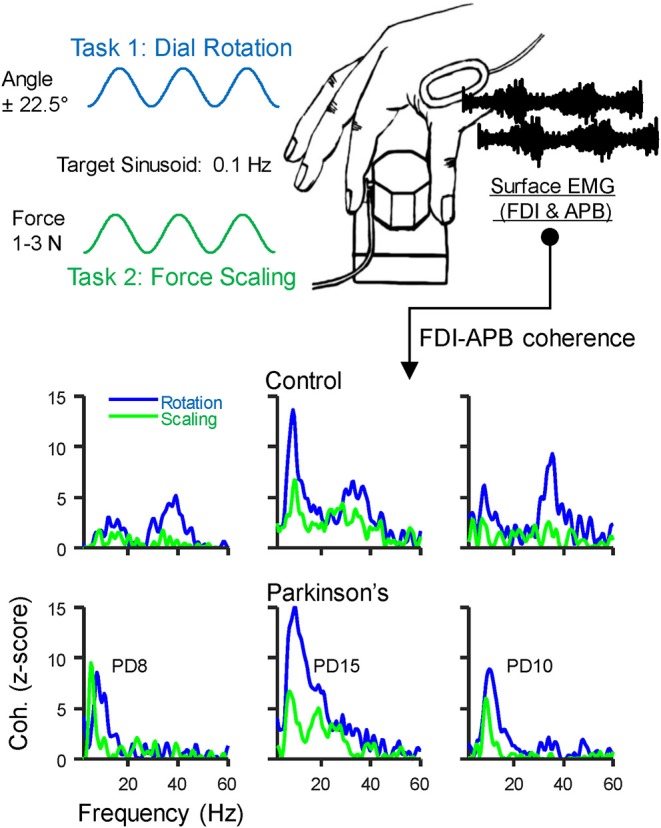
Behavioral Task: Participants grasped a small dial between the thumb and index finger and either (1) slowly scaled their pinch force using visual feedback to track a 0.1 Hz sinusoid spanning 1–3 N, or (2) rotated the dial back and forth over 45 degrees at 0.1 Hz using visual feedback of dial rotation. A miniature load cell at the index finger pad measured pinch force, and surface EMG was recorded over the first dorsal interosseous (FDI) and abductor pollicis brevis (APB) muscles. The EMG signals were used to calculate intermuscular coherence. The bottom panels show coherence profiles for each task from three example individuals in the control group (top) and in those with Parkinson's disease (PD, bottom). The displayed identification numbers correspond to the participant descriptions in Table 2.

We asked participants to pinch or rotate a custom-made dial (diameter: 3 cm) between the thumb and index finger, as described in Laine and Valero-Cuevas (19) (see Figure 1). Both tasks generate a physiological tremor in the muscles of the thumb and index finger, and coherence between their EMG signals varies across tasks even without related changes in the shape of their power spectra. Therefore, these tasks alter the extent to which alpha-band neural drive is shared among muscles rather than simply altering its amplitude. Briefly, the dial held a potentiometer to track rotation angle and a miniature load cell (ELB4-10, Measurement Specialties, Hampton, VA) under the index finger to measure pinch force. Surface EMG sensors (Biometrics, Newport, UK) were placed over the first dorsal interosseous (FDI) and abductor pollicis brevis (APB) muscles of each hand. All signals were acquired at 1,000 Hz using a Biometrics LTD DataLINK system (Biometrics, Newport, UK). Visual feedback of rotation or force was provided using custom software designed in MATLAB (The Math Works, Natick, MA). We instructed participants to prioritize the production of a smooth force or rotation effort, guided/paced by the target sinusoid. This instruction was intended to reduce the likelihood that participants with involuntary tremors would focus on counteracting them rather than executing the prescribed slow voluntary action. Each participant completed four, 3-min tasks with each hand, (2 trials for each of 2 tasks, described below). Practice trials were given prior to recordings, and breaks between each task were given to prevent fatigue. The order of tasks and hands was randomized for each participant, and subjects did not find these simple tasks fatiguing.

Task 1: Dynamic modulation of isometric pinch force. With each hand separately, participants pinched the dial and slowly varied their pinch force between 1 and 3 N by tracking a sinusoidal target displayed on screen. The vertical height of the cursor was controlled by pinch force and while the horizontal position moved left-to-right across the screen automatically as a function of time, taking 30 s before looping back to the left. The sinusoidal target had a peak-to-peak period of 10 s, such that the frequency of force modulation was 0.1 Hz. We chose this frequency for ease of tracking and used it for all participants to avoid potential effects of movement speed on our EMG measurements. Practice trials were given to familiarize each participant with the task prior to recordings.

Task 2: Dial rotation. In this task, subjects rotated the dial back and forth +/– 22.5 degrees with each hand separately. Visual feedback was provided as before, but with the vertical position of the cursor controlled by rotation angle. To ensure that pinch force remained similar across tasks, the cursor color changed to indicate if pinch force exited the 1–3 N range during the task. All participants were able to maintain pinch force within this range and made few errors after initial practice.

EMG signals were high-pass filtered using a zero-phase 4th order Butterworth filter with a cutoff at 250 Hz, then rectified and normalized to unit variance as in our previous study (19). This follows the general recommendations for accentuating the timing and density of motor unit action potentials within the surface EMG signal (43–47). It should be noted that the extreme high-pass filtering may be a precaution more than a universal necessity, and that surface EMG signals can be expected to contain some degree of noise or amplitude cancellation which can distort coherence measures when assessing low frequency neural drive (e.g., <5 Hz) or using high contraction levels (48), neither of which are a concern in the present study. The two trials for each task for each hand were concatenated, yielding a total of 6 min of data per hand. The first few (~5) seconds of each trial were trimmed manually to ensure that stable tracking had been obtained prior to analysis.

### Coherence Analysis

Coherence between EMG signals describes the frequency content of their synchronized activity. Coherence between the EMG signals of the FDI and APB muscles was calculated using the “mscohere” function in MATLAB, specifying segment sizes of 2 s, tapered with a Hann window, and overlapped by 0.5 s. Prior to statistical comparisons, the raw coherence values (C) were first converted to Fisher's Z values using the formula *Fz* = atanh [sqrt(C)]. Then, for better comparison with previous work, and to provide a more standard index of statistical strength, we converted the *Fz* values to standard Z-scores using the formula *Z* = Fz / √ (1/ 2L)—bias. In this formula, L is the degrees of freedom derived from the number of segments used in the coherence calculation (49–51), and the bias was calculated as the mean uncorrected Z-score between 100 and 500 Hz, since this frequency range contains no physiological coherence (19, 52). Coherence profiles for three individuals from each group are shown in the bottom panels of Figure 1.

### Statistical Testing

Statistical evaluation of coherence is often simplified by binning the frequency spectrum into a few common bands of interest. The exact boundaries for each band can vary across studies, and such boundaries may change when evaluating pathology. Therefore, to address our main hypotheses, we allowed relevant frequency bands to be defined from the data itself, using a non-parametric version of statistical parametric mapping (SPM), as described previously (53, 54). This is a random-permutation test that assigns *p*-values to regions of interest (corrected for multiple comparisons) within a “map” of cross-group differences calculated over space, time, or (in our case) frequencies (55). To map group differences in coherence across frequencies, we used an effect size measure, Cohen's D, as our initial statistic. This was then smoothed over frequencies using a gaussian window spanning 4 Hz. We defined regions of interest, or “clusters,” as any group of consecutive frequencies (min width = 3 Hz) exceeding a threshold. The threshold can be set arbitrarily but we automated this by using the 95% confidence interval for our Cohen's D values, calculated with respect to their mean and standard deviation from 100 to 500 Hz, where no true group level differences in coherence should exist. The above-threshold area of each cluster was tested for statistical significance based on a 10,000 iteration random permutation test, as described previously (55).

Using the above procedure, we identified differences in intermuscular coherence between controls and participants with PD for (1) the rotation task, (2) the scaling task, and (3) the difference between rotation and scaling (rotation-scaling, per hand). The latter addresses whether any effects of PD on intermuscular coherence are task-specific. For each, we also created box and whisker plots to visualize how the average coherence within each statistically significant frequency band varied across individuals. To better assess variability across individuals rather than hands, data from both hands were averaged per individual. A Cohen's D effect size as well as a *p*-value (derived from a random-permutation test) were also calculated for this binned/averaged data. Since it is possible that PD may have stronger effects on one hand, we confirmed the appropriateness of averaging across hands by calculated an absolute laterality index for each individual (the absolute difference in coherence between hands divided by their sum), and comparing across groups, again using a random-permutation test.

### Correlation Analysis

The intermuscular coherence values above were tested for correlation with the total UPDRS score, as well as with its subsection II (activities of daily living) and subsection III (motor evaluation). To be conservative, this was conducted using a non-parametric Spearman's rank correlation. The correlation coefficients and associated *p*-values were obtained using the “corr” function in MATLAB. This analysis tested for a non-zero correlation between coherence and symptom measures. With so few participants, an exact magnitude of correlation cannot be determined with high precision. We therefore calculated a 95% confidence interval around each correlation measure using a 10,000 iteration bootstrap procedure.

### Discriminability

We evaluated the extent to which the data above could classify a participant as being a member of the PD or control group. To do this, we constructed Receiver Operating Characteristic curves (ROC curves) for each task [for a brief overview, see Eng (56)]. Each point on an ROC curve describes the fraction of patients who could be correctly identified (true positive rate, y-axis) using a particular threshold value for discrimination, while at the same time misclassifying some proportion of the controls (false positive rate, x-axis). Defining these proportions for every possible threshold produces the ROC curve. The area under the curve (AUC) is 1 for perfect discrimination and 0.5 for random chance. Our SPM analysis identified that the greatest difference of coherence between PD and control groups occurred in the alpha-band for both tasks (5.8–16.6 Hz for the rotation task and 4.4–15.6 for the scaling task). Using this information, we extracted a single measurement from each coherence spectrum, herein referred to as the alpha-ratio, by calculating the fraction of total intermuscular coherence (between 2 and 80 Hz) falling within the alpha-band (defined per task, as described above). This ratio-based normalization strategy has been used previously for reporting tremor measures (23, 57), and in our case, it reduces inter-subject variability from sources that could influence all frequencies at once (e.g., noise or cross-talk) while emphasizing the overall shape of the coherence spectrum. ROC curves were first constructed for each task. Then, for a final combined analysis, we created an ROC curve after averaging all 4 alpha-ratios obtained for each participant (2 hands × 2 tasks).

### Task Performance

All participants could execute the task with both hands. Because systematic differences in the overall frequency/magnitude of error corrections could influence alpha-band drive to individual muscles (58, 59) we used a 10,000 iteration permutation test to determine if fluctuations in force/rotation about the target sinusoid differed significantly between groups. Here, performance was quantified as the standard deviation of pinch force or rotation angle after filtering out the 0.1 Hz modulation associated with the voluntary task of tracking the target sinusoid. Task performance was also tested for correlation with alpha-band drive using Spearman's rank correlation.

## Results

**Figure 2:**
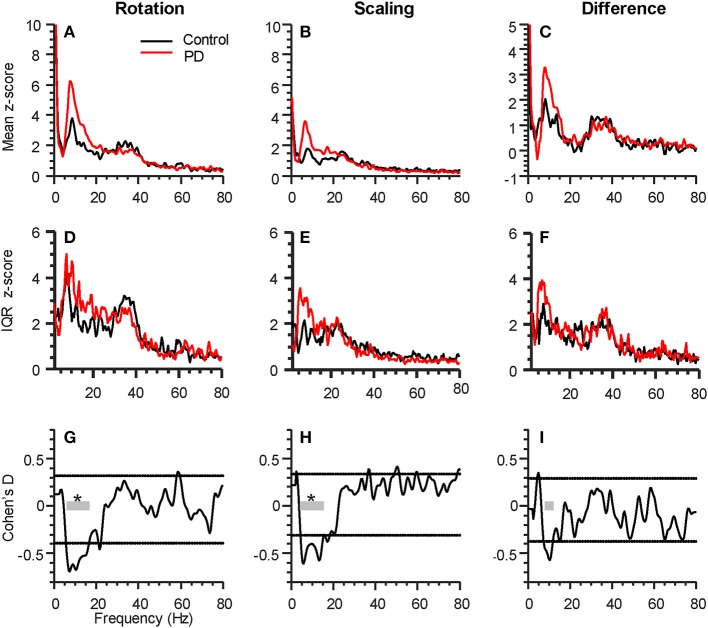
Parkinson's disease (PD) affects intermuscular coherence in the alpha-band (6–15 Hz). The three columns represent analyses of group differences in intermuscular coherence (Control, black, 15 participants; Parkinson's, red, 16 participants) during the rotation task (left), the force scaling task (middle), as well as the difference between the two tasks (rotation minus scaling, right). All plots include data from both hands of each participant. **(A–C)** shows the grand average FDI:APB coherence in each task. **(D–F)** shows the interquartile range (IQR) of coherence z-scores across subjects at each individual frequency sample. **(G–I)** shows the statistical difference between groups (expressed as Cohen's D), calculated at every frequency. The horizontal dashed lines represent a statistical threshold for identifying frequency bands of interest for further statistical testing (see Methods). The gray bars show the bands of interest identified for each condition, with an * indicating that the band as a whole differed significantly between groups (*p* < 0.05, corrected for multiple comparisons).

For both tasks, alpha-band coherence between the FDI and APB muscles differed between the PD and control group. For the rotation task, coherence differed within the frequency range of 5.8 to 16.6 Hz, with *p* < 0.001 (see Figures 2A,G). The scaling task showed significant difference between groups from 4.4 to 15.6 Hz, with *p* < 0.001 (Figures 2B,H). The difference between the two tasks (rotation—scaling) showed a range of interest between 7.8 and 12.2 Hz (Figures 2C,I), but was not statistically significant (*p* = 0.58). Intersubject variability was generally high (Figures 2D–F).

**Figure 3:**
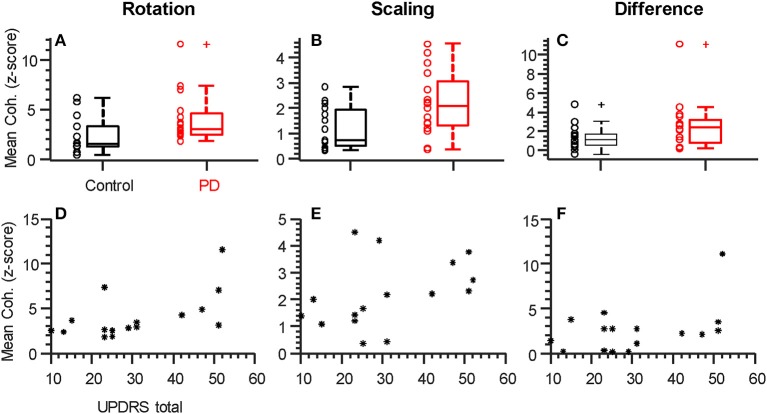
Individual alpha-band coherence profiles differ between groups and correlate with UPDRS. **(A–C)** Box plots summarizing the mean coherence values for each participant calculated over the frequency range of significant difference (both alpha-band, see Figure 2). Both rotation **(A)** and scaling **(B)**, but not the difference between tasks **(C)**, showed significantly (*p* < 0.05) larger values in the Parkinson's Disease group relative to controls. **(D–F)** Total UPDRS scores vs. mean coherence (as plotted above). The correlation between UPDRS scores and alpha-band coherence was strongest for the rotation task and statistically significant (*D*, rho = 0.058, *p* = 0.018, see Table 2). + symbols indicate outliers. ° symbols show the individual data from each participant, as summarized by the box plots. * symbols show individual data from each participant with PD.

Figures 3A–C show the consistency of alpha-band differences in intermuscular coherence between controls and participants with PD, for each task separately, as well as and their difference. For these plots, the coherence values are averaged across both hands per individual, since we found that there were no group differences (PD vs. CT) in the laterality of coherence between right and left hands (*p* = 0.55, *p* = 0.36, and *p* = 0.57 for rotation, scaling, and their difference, respectively). For consistency, we defined the alpha-band per task according to the precise frequency ranges identified in our SPM analysis. Using a generic 6–15 Hz frequency range for all tasks produced nearly identical results. Generally, a substantial degree of overlap between groups was apparent due to high inter-subject variability. Nonetheless, this binned/averaged alpha-band coherence measure showed significant differences between groups (*p* = 0.04, *p* = 0.02) for rotation and scaling tasks, respectively. Within each group, coherence in the alpha-band was task-dependent, differing significantly between scaling and rotation tasks (*p* = 0.008 and *p* = 0.005 for control and PD groups, respectively), but again, the magnitude of this difference did not differ between groups (*p* = 0.95). Coherence differences between groups had Cohen's D effect sizes of 0.75, 0.91, and 0.58, for the rotation task, the scaling task, and their difference, respectively.

Within the PD group, it is possible that coherence within the alpha-band covaries with symptom severity. We therefore tested the hypothesis of a non-zero correlation between coherence (as plotted in Figures 3A–C) and the total UPDRS score for each individual (Figures 3D–F and Table 2). Table 2 shows the Spearman correlation coefficients between coherence in the alpha-band and the total UPDRS score, as well as the two main subsections of the UPDRS score: the motor symptoms score (Part III), and the self-evaluation of daily activities (part II). The 95% confidence interval around each correlation value is shown as well. Moderate correlations were found for coherence within the rotation task but not the force scaling task or their difference. Overall, the correlation between intermuscular coherence and symptom severity was not restricted to (or strongest for) the motor-only portion of the scale (UPDRS III), suggesting a more general relationship with disease state.

**Table 2:** Spearman's correlation (rho) between mean coherence within the alpha-band, and patient symptom severities, as per the UPDRS, along with 95% confidence interval (CI) boundaries.

	**UPDRS total**	**UPDRS II**	**UPDRS III**
Rotation	**rho** **=** **0.58**	rho = 0.48	**rho** **=** **0.50**
	**CI** **=** **[0.11 to 0.92]**	CI = [−0.04 to 0.82]	**CI** **=** **[−0.03 to 0.84]**
	***p*** **=** **0.018**	*P* = 0.058	***p*** **=** **0.046**
Scaling	rho = 0.467	rho = 0.46	rho = 0.40
	CI = [−0.03 to 0.82]	CI = [−0.04 to 0.81]	CI = [−0.05 to 0.72]
	*p* =0.068	*p* = 0.073	*p* = 0.123
Difference	rho = 0.26	rho = 0.35	rho = 0.28
	CI = [−0.28 to 0.7]	CI = [−0.24 to 0.81]	CI = [−0.30 to 0.72]
	*p* = 0.327	*p* = 0.186	*p* = 0.293

*Correlations were found to be significantly non-zero (bold, p < 0.05) between the rotation task and the total UPDRS score, and to a lesser extent, with the UPDRS motor-only score*.

To determine if the accuracy with which controls and participants with PD performed the visuo-motor tracking related to coherence metrics, we compared the standard deviation of rotation angle or pinch force (after removing the 0.1 Hz target frequency), separately across groups. Task performance did not differ across groups (*p* = 0.67 and *p* = 0.86, respectively). This implies that any changes in alpha-band neural drive did not substantially contribute to tracking error, and indeed, neither group showed a significant correlation between tracking performance and alpha-band coherence in either task.

**Figure 4:**
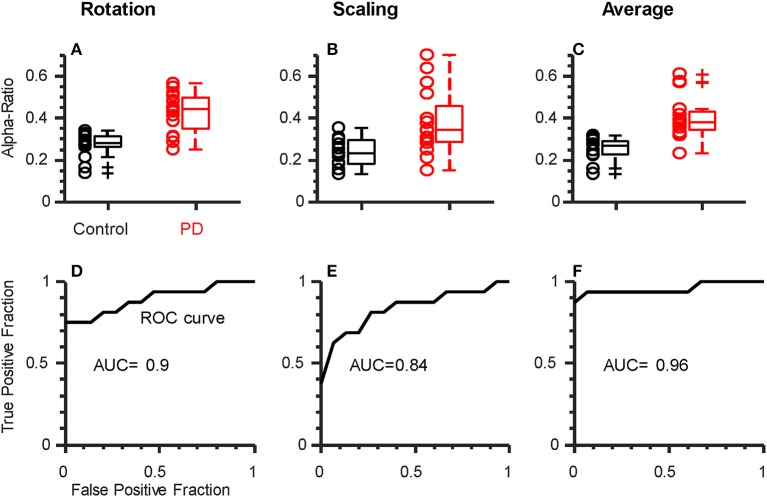
Alpha-ratio (i.e., proportion of total intermuscular coherence within the alpha-band) can discriminate participants with PD from controls. Top row: Box plots showing the average alpha-ratio (across hands) for each individual. Results are shown for rotation **(A)**, scaling **(B)**, individually, as well as their combination **(C)**. Both rotation **(A)** and scaling **(B)** showed reasonable separation between groups, but the combination of both (average) per individual resulted in the least overlap **(C)**. **(D–F)** Receiver Operating Characteristic (ROC) curves for the metrics shown above. Area Under the Curve (AUC) values indicate strong-to-excellent discriminability in all cases.

Finally, we sought to determine if a single coherence index calculated for each individual could be used to discriminate between groups. For this analysis, we calculated the proportion of total coherence (2 to 80 Hz) falling within the alpha-band for each task/hand (the “alpha-ratio”). This is a simple way to reduce inter-subject sources of variability and focus on the contribution of alpha-band coherence to the overall shape of the coherence spectra. We confirmed that the alpha-ratio showed no group differences in laterality between hands (*p* = 0.41 and *p* = 0.8 for rotation and scaling tasks, respectively), and therefore averaged across hands to obtain a single ratio for each individual, per task. Figures 4A,B shows a clear separation between PD and control groups for the rotation (*p* < 0.001, Cohen's D = 1.96) and scaling task (*p* = 0.001, Cohen's D = 1.22). Since the purpose of this test was to determine the potential for coherence measures to discriminate between groups, we did not include an evaluation of the change in coherence between tasks, as there were no group differences in this measure. Instead, we averaged the alpha-ratio values obtained from the scaling and rotation tasks together (Figure 4C) to obtain a single, combined alpha-ratio per individual, which also differed significantly between groups (*p* < 0.001 Cohen's D = 1.84). We constructed Receiver Operating Characteristic Curves for the alpha-ratios derived for each task and their combination (Figures 4D–F). To quantify the overall discriminability, we calculated the area under the curve (AUC), which yielded values of 0.9, 0.84, and 0.96 for rotation, scaling, and their combination, respectively. These high values indicate that excellent separation between groups was possible in our study population, and that nearly all patients could be correctly classified, with practically no false positives (misclassified controls).

## Discussion

In this study, we describe a robust and consistent PD-related amplification of alpha-band (~6–15 Hz) intermuscular coherence between finger muscles evoked during simple dynamic actions. Slow rotation of a dial between the thumb and index finger produced the strongest coherence, but the same effect was also observed during isometric modulation of precision pinch force. Clinical ratings of symptom severity (as per UPDRS) correlated significantly with the increased magnitude of intermuscular coherence during the rotation task only. The global alpha-ratio (proportion of total coherence within the alpha-band) provided an index which allowed excellent discrimination between controls and participants with PD within our study sample. This provides valuable information for future development of simple, practical measures of neural dysfunction in PD.

### Kinetic Action Tremor in PD

The disruptions in neural drive that we have characterized in this study appear to be an intermuscular component of Parkinsonian kinetic action tremor (1, 4, 23, 60). In 1963, Lance et al. noted anecdotally that in PD a 5–15 Hz tremor was (i) always present at the beginning of a muscle contraction, (ii) was sometimes sustained during the static portion of a contraction, and (iii) was usually visible within EMG traces as a synchronous “grouping” of action potentials within and across contracting muscles—even when the tremor itself was not detectable by eye. Action tremor is distinct from rest tremor [or its re-emergence during steady contraction (2)] in that is higher in frequency (6–15 vs. 3–5 Hz), enhanced during dynamic action rather than reduced, and it is not attenuated by dopamine replacement in PD (4).

There is currently no standard clinical procedure for quantifying 6–15 Hz action tremor, perhaps because it is often invisible without special equipment and can be difficult to distinguish from “normal” physiological tremor (discussed further below). Most previous literature quantifies the magnitude of tremulous activity at a particular muscle or joint [e.g., (4, 60–63)]. However, if action tremor stems from an inherently common neural drive to multiple muscles, then it makes sense to use intermuscular coherence to quantify it and avoid reliance on limb/task/person-specific forces and motions.

Although the idea that tremor-generating neural drive in PD is inherently distributed to multiple muscles has received some support (21, 22), there has been less effort to identify the particular muscles/tasks in which an intermuscular action tremor is most reliably evoked and detected. So far, an amplified intermuscular coherence within the alpha-band has been reported only for static tasks such as isometric knee extension (11) or across two sides of a puckered upper lip (7). However, the alpha-band drive underlying action tremor in PD is best observed during dynamic actions (1, 4), where additional neural control circuitry may be required (64). Also, previous findings have described amplified coherence across anatomically synergistic muscles which naturally share a substantial amount of their neural drive (17). This makes it difficult to disambiguate changes in overall signal strength from an effect of PD on the process of “binding” of muscles into functional synergies through neural synchronization (65). Our results seem to suggest that a natural intermuscular alpha-band drive is amplified in PD. Further, the modification of this drive across different tasks is preserved, indicating that the amplification is not especially task-specific. This may indicate that the neural circuit which distributes alpha-band drive across muscles is still functional, even if overdriven.

### Neural Origins of Parkinsonian Kinetic Action Tremor

Action tremor has been described as an amplification of naturally present physiological tremor rather than something that exclusively emerges in PD (4, 60); a notion that is well-aligned with our current data. Because the origins of physiological tremor are likely multifactorial (27–36), the effects of PD on each potential source of alpha-band neural drive to muscles must be considered. There are at least four potential sources for alpha-band neural drive in healthy individuals, and they are not mutually exclusive: (1) the stretch reflex system, (2) the motor cortex, (3) the brainstem (specifically reticulospinal output), and (4) the cerebellum (via the cerebello-thalamo-cortical circuit). We favor the latter, according to the following rationale.

If alpha-band drive is measured within the output of a single muscle, it may simply reflect cycles of excitation around the monosynaptic Ia afferent reflex loop of that muscle (27, 33, 34, 58, 66). However, this mechanism would not easily explain coherent ~10 Hz activity across different muscles, which can be observed even between muscles in different hands in healthy adults (32, 38). Mechanical coupling among muscles may serve to link afferent feedback without need for monosynaptic connectivity, and a potential role for spinal interneuron pools in coordinating afferent activity across multiple muscles cannot be ruled out, but to our knowledge, there is no evidence that either mechanism could explain the generation of intermuscular coherence between the FDI and APB within the context of our task. Also, an origin in the peripheral reflex system would imply that stretch reflex or H-reflex amplitudes should be elevated in PD, which is not the case (67, 68). PD can, in some cases, disrupt task-dependent stretch reflex modulation (68), suggesting upstream mismanagement rather than pure peripheral dysfunction.

Studies of neural drive to individual muscles have indicated potential cortical involvement in alpha-band Parkinsonian tremor (12–18 Hz) during steady contraction of the wrist extensors (69, 70), although a subsequent delay analysis suggested that the cortical activity might not generate this drive, but instead, it may receive an efference copy from a subcortical source (69). Also, steady muscle contractions do not usually produce alpha-band corticomuscular coherence (EEG-EMG), even when intermuscular coherence (EMG-EMG) in this range is simultaneously observed (38, 71). For these reasons, it seems unlikely that our findings reflect an amplification of corticospinal output.

The reticulospinal pathway may also produce an alpha-band drive to muscles. Acoustic startle stimulates the reticulospinal pathway (72) and produces a brief wave of 12–16 Hz (73) coherent activity in bilateral pairs of muscles. However, involuntary reactions to acoustic startle may not directly predict reticulospinal contributions to intermuscular coherence during voluntary tasks. The potential association between acoustic startle responses and intermuscular coherence during voluntary tasks has never been fully explored, and current evidence is indirect. For example, spasticity in chronic stroke is thought to relate to overdriven reticulospinal drive to muscles (74, 75), and both alpha-band intermuscular coherence (76, 77) and responses to acoustic startle are increased in spastic muscles of stroke survivors (78, 79). However, if PD amplifies reticulospinal output, startle responses should be increased in magnitude or consistency relative to controls, but they are not (80). The preparation of brainstem output by upstream circuits may, however, be abnormal in PD since acoustic startle does not produce the usual acceleration of reaction times (the “StartReact” paradigm) in people with PD-associated freezing of gate (81). Therefore, the amplified alpha-band drive in PD may involve reticulospinal pathways, but probably not through an overall change in the excitability of the pontomedullary reticular formation.

Finally, the frequency spectrum of neural drive to muscles may be substantially influenced by the cerebellum via its actions within the cerebello-thalamo-cortical (CTC) circuit. The cerebellum oscillates in synchrony with the motor cortex at frequencies between 10 and 40 Hz (82), and slow movements of the finger produce coherent alpha-band oscillations between muscle activity and the cerebellum, thalamus, and motor cortex (30). In addition to a possible influence on the cortex and corticospinal output to muscles, the deep cerebellar nuclei may also transmit a 10 Hz oscillation to muscles through modulation of the brainstem and reticulospinal tract output (82–84). In theory, output from the deep cerebellar nuclei to the cortex and brainstem could be amplified (disinhibited) in PD since Purkinje cell damage is associated with the disease (42, 85, 86). Regardless of the precise pathway to muscles, a wide variety of studies have implicated general CTC dysfunction in PD (40–42, 87–89). Further, dysfunction of the CTC is consistently associated with the generation of tremor in both PD (90–92) and the more common, but often-associated (93) condition known as essential tremor (94–99). In fact, medication in PD may reduce rest tremor by reconnecting cerebellar communication with the cortex through the ventrolateral thalamus (92), and a similar mechanism may explain the levodopa-reversible loss of 40 Hz corticomuscular drive in PD (12), since inactivation of the cerebellar cortex disrupts gamma-band cortical activity (100). In this view, basal ganglia dysfunction, as in PD, almost inevitably implies cerebellar network dysfunction. In fact, these structures have been described recently as nodes within a larger, tightly-interconnected network (101).

Taken together, it is clear that damage to this cerebellar circuit is not only plausible within our study population, but would very likely produce changes in alpha-band drive to muscles. Given the strong associations between cerebellar activity, cortical activity, and alpha-band spindle discharge during slow movements (102), it is likely that cerebellar-circuit dysfunction could both directly and indirectly influence the frequency spectrum of neuromuscular oscillations in PD. In addition to shaping neural oscillations, cerebellar circuit dysfunction can be expected to influence a variety of non-motor functions as well (86, 91, 103–106) and this might explain why correlations between coherence and UPDRS scores were not restricted to the direct motor evaluation in our study. That said, a specific or exclusive role for the CTC in the present study not possible to determine, and will require further research.

### Intermuscular Coherence as a Potential Clinically-Practical Biomarker in PD

We found that the proportion of total coherence within the ~6–15 Hz range was sufficient to statistically separate groups in either task. When all ratio values obtained from each individual were averaged together, it allowed for excellent discrimination between our two groups (AUC of 0.96). If this level of discriminability were to generalize to the larger population, it would meet or exceed the performance of the best MRI-based diagnostic biomarkers to date (107, 108) and yet not require expensive neuroimaging, invasive collection of cerebrospinal fluid, extensive patient history, etc. To be clear though, much larger studies would be needed to evaluate the readiness of any putative biomarker for clinical translation. Our intent in exploring discriminability between groups was not to establish an application-ready feature set or threshold for optimal real-world classification, but rather to provide evidence that EMG-based measures have clinical potential, since they have rarely been explored in this capacity. In fact, the only other EMG-derived measure which has achieved such strong discrimination between PD and controls was calculated from the cross-trial distribution of EMG burst durations in the biceps during fast elbow flexions in temporarily-off-medication patients (57). The neural mechanisms that contributed to that effect were not identified, and the measurement equipment and procedure itself was somewhat specialized and not well-suited for widespread clinical implementation. Our simpler method, with further refinements, seems more practical for common application and to complement other non-invasive biomarkers, such as those derived from olfaction (109, 110), or finger movements (111, 112).

### Limitations

An important consideration in this study is that we recorded from participants who were on their normal medication, as this was a sample-of-convenience. To our knowledge, there is no evidence that medication could explain the increased alpha-band neural drive we observed, especially since tremor in this frequency range is not enhanced or attenuated by medication (4, 113). Of course, if it were attenuated, then presumably unmedicated participants would show even stronger differences from controls, strengthening our classification ability. Medication may have normalized 30–50 Hz intermuscular coherence in our study, as this frequency band is dopamine-dependent (12), but again, if our analysis were applied to unmedicated participants we would expect better discrimination between PD and controls, not worse. In PD, dopamine depletion in the basal ganglia is already extreme by the time of symptom onset (e.g., ~80% loss in the putamen) making it difficult to detect or study the early stages of the disease (114). Thus, a method capable of detecting both low dopamine and amplified physiological tremor could contribute to such efforts, especially if combined with PD-sensitive metrics of finger movement speed/timing (111, 112). This would, in turn, enable the early initiation of neuroprotective strategies once they become available. Of course, any claims concerning translational/diagnostic capabilities at this point is speculative. Our sample size does not allow us to accurately characterize the strength and variability of effects across the total population of people with PD. Effect size estimates such as Cohen's D may be inflated in smaller studies, while skewed distributions or outliers may have the opposite effect. That said, our study is appropriately powered to detect the strong effects that are most likely to be of scientific and translational relevance, as well as to justify/enable their investigation in larger cohorts. Additionally, determining the optimal battery of task parameters will require future work. For example, it may be that features of visual feedback, the speed of the required dynamic action, the particular muscles measured, etc. may all be important variables. Also, this study focused on kinetic action tremor, however, the postural form of action tremor which emerges during static contractions may yield complementary information. A final limitation regarding intermuscular coherence analysis is that it measures the relative proportion of shared vs. total EMG signal variance, and thus changes to both intramuscular (muscle-specific) and intermuscular (shared) neural drive can alter coherence. Separation of shared and muscle-specific neural drive can be accomplished using methods based on motor unit analysis [e.g., Laine et al., (17)], and may represent a valuable direction for future investigation. At present though, it is clear that tasks designed to evoke kinetic action tremor from functionally-coordinated muscles provide a clear window into the nervous system, which holds value for the assessment of PD and its progression.

## Data Availability Statement

The datasets generated for this study are available on request to the corresponding author.

## Ethics Statement

The studies involving human participants were reviewed and approved by University of Southern California Internal Review Board. The patients/participants provided their written informed consent to participate in this study.

## Author Contributions

CL and FV-C contributed to the study design, interpretation of data, editing, and approval of the final manuscript. CL contributed to collection of experimental data, data analysis, and drafting of the manuscript.

### Conflict of Interest

The authors declare that the research was conducted in the absence of any commercial or financial relationships that could be construed as a potential conflict of interest.
